# Eastern Ohio Dental Association

**Published:** 1872-03

**Authors:** D. B. McLain


### EASTERN OHIO DENTAL ASSOCIATION.

ALLIANCE, OHIO, Feb. 27, 1872.

The second semi-annual meeting of the Association was convened at 1 o'clock by the President, Dr. J. C. Whinery of Salem, who invited the Rev. Mr. Wolf, of Alliance, to open with prayer. Members present, Whinery, Lyder, McLain, Douds, Siddal, Porter, Hole and Hisey.

The Treasurer reported having in his hands $7.80.

Dr. J. M. Porter, of Massillon, read a very able and elaborate essay on anæsthesia and respiration.

Dr. J. W. Lyder, of Alliance, read an essay on Dental Association and its benefits, pointing to the great advance made through the influence of Dental Associations, and alluding to the damaging influence on the profession of those men who do not believe in association, or who attend one meeting for pecuniary motives only, in order to class themselves as members in good standing. On motion, these views were adopted as the sentiment of the Association.

Dr. Siddal gave his method of producing anæsthesia, using chloroform and ether. Dr. Whinery addressed the Association on anæsthesia. Dr. Lyder briefly deprecated the, in many cases, low fees for administering anæsthetics, taking into consideration the great danger always attendant. Dr. Siddal delivered an oration on the rise and progress of the dental profession and laudatory of the shining lights of past days.

On motion, a cordial invitation was given to members of the medical profession present to participate in the discussion on the subject, "When is extraction indicated". Dr. Douds, of Canton, always extracts the second bicuspid in cases where the cuspid is crowded out of the arch, providing, extraction of any tooth is necessary and the first bicuspid is sound, as from observation he thinks the first bicuspid less subject to decay. Dr. McLain reported cases where he had successfully treated excessive deformity of the mouth, resulting from irregularity of cuspids, by extracting the first bicuspids, preferring them on account of being able to bring the cuspids into place with less disturbance than by extracting any other tooth. If there was not room sufficient procured it by plates or wedging.

Dr. Porter condemned in strong terms the wholesale extracting of diseased teeth and roots, claiming that in most cases they can be restored to health by judicious treatment.

Dr. Lyder reported a case where a superior lateral incisor, perfectly sound, closing inside the arch, received such injuries from unnatural occlusion, as to cause alveolar abscess, the root of the tooth when extracted being almost jet black.

Dr. McLain reported a case where a lady, age forty five, after the extraction of the entire upper denture, erupted a molar near the mesian line, about six lines posterior to the natural position of the right lateral incisor, the side of the tooth presented to the mouth, the roots pointing forward, and a bicuspid in the natural place and position. Also a case where one of three sisters had an entire denture extracted, and the other two the upper denture, not one tooth decayed, but all dead, and a source of constant irritation and inflammation. Most of the roots were necrosed, cause a depraved condition of the general system.

Dr. Whinery reported several cases of complicated disease of Maxillary Sinus treated successfully with diluted chloride of zinc and diluted carbolic acid, alternately, one case being of many years standing.

On motion, the next place and time of meeting was appointed at Salem, August 29th, 1872, 10 A. M.

A committee consisting of Drs. Coffee, Douds and Hisey, was appointed to nominate delegates to the American Dental Association They selected Drs. Porter, of Massillon; Whinery, of Salem; and McLain, of New Lisbon, who were thereupon elected by the Association.

The Chair appointed as essayists for the next meeting, Drs. Porter and Douds. Subjects for discussion, "The proper treatment and filling of diseased teeth and roots," "Dental Ethics," "Specialties in Dentistry."

The following resolution by Dr. Whinery was adopted:

*Resolved*, That this Association heartily endorse the action of the State Dental Association in appointing a committee to defend the dentists of the State against the demands of the Dental Vulcanite Company under the Cummings patent.

After thanking the Masonic Order for the use of their hall, the Association adjourned.

D. B. McLain, Secretary.

